# TEAMWORK MAKES THE DREAMWORK: CHANGES IN INTERPROFESSIONAL READINESS

**DOI:** 10.1093/geroni/igad104.3620

**Published:** 2023-12-21

**Authors:** Ann Rhodes, Kristin Zimmerman, Sarah Marrs, Leland Waters

**Affiliations:** Virginia Commonwealth University, Richmond, Virginia, United States; Virginia Commonwealth University, Richmond, Virginia, United States; Virginia Commonwealth University, Richmond, Virginia, United States; Virginia Commonwealth University, Richmond, Virginia, United States

## Abstract

Creating Interprofessional Readiness for Complex & Aging Adults (CIRCAA) is an evidence-based program grounded in the 4Ms of age-friendly care. CIRCAA aims to improve care for older adults by enhancing Scholars’ ability to affect patients, learners, and team members by emphasizing interdisciplinary collaboration in person-centered care. Among other assessments, Scholars assess their interprofessional skills using the interprofessional education competency (IPEC) tool at the beginning and end of the year-long program. Evaluation data from all four CIRCAA cohorts (2020-2023; n = 22) demonstrated significant improvement in interprofessional readiness; t(34) = -4.72, p < .001. The sample across years was well-balanced; there were no significant differences in size of cohort {F(1) = 0.516, p = .47}, race {F(3) = 0.62, p = .6}, or discipline and clinical practice of Scholars {F(1) = 0.33, p = 0.56}. There was a significant difference in knowledge gained based on years of experience working with older adults {F(4,26) = 3.308, p = .025}; those who had worked with older adults for less than five years made the largest gains. Scholars working in primary care settings had larger knowledge gains than Scholars who did not work in primary care, with gains of 4.95 points and 7.87 points, respectively. This difference was not statistically significant; t(16.3) = 1.14, p = 0.2, 95% CI [-2.49-8.32]. Overall, assessment of outcomes across the four years shows substantial gains in interprofessionalism, which are particularly pronounced in new clinicians and those working in primary care settings.

